# Potent Inhibitory Activity of Natural Product Anaephene B and Analogues against *Leishmania tarentolae* In Vitro

**DOI:** 10.3390/molecules28030946

**Published:** 2023-01-18

**Authors:** Shariq M. Zaman, Marjorie A. Jones

**Affiliations:** Department of Chemistry, Illinois State University, Normal, IL 61790-4160, USA

**Keywords:** natural product anaephene B, analogues, *Leishmania tarentolae*

## Abstract

In this study, a specific alkylphenol natural product, anaephene B, and its unique synthesized derivatives were tested for their inhibitory effect on the protozoan parasite *Leishmania tarentolae*. In a series of cell viability tests and enzyme assays, these test compounds have produced interesting results with regard to their antibiotic effect, showing similar potency against *L. tarentolae* as they do against drug-resistant bacteria such as methicillin-resistant *Staphylococcus aureus* (MRSA). All compounds tested in this study have shown the ability to completely inhibit our model system, *L. tarentolae,* in vitro. This study helps increase our understanding of the structure-activity relationship (SAR) between anaephene B and its analogues for a new class of potential pharmaceuticals for the treatment of *Leishmania* infections.

## 1. Introduction

*Leishmania* are single-celled protozoan parasites that are responsible for infecting various species, including reptiles and mammals [1]. These parasites are transmitted through the bite of infected female phlebotomine sandflies, leading to a disease known as leishmaniasis. Predominately in developing countries, this disease continues to affect humans, leading to symptoms such as ulcers in the skin and mucous membranes and/or damage to internal organs [2]. There is a significant need to develop new pharmaceuticals that have increased effectiveness and fewer side effects than drugs, such as pentavalent antimonials and amphotericin B, currently being used [3,4,5]. In addition, drug resistance to these is being reported [6,7].

To treat many diseases, scientists have found antibacterial natural products very useful in the development of therapeutics. Natural products are chemicals that are made by some biological systems affording them an advantage over other organisms. Due to their biological properties and their wide structural diversity, these natural products provide a good basis for the synthesis of new antibiotics [reviewed by Cowan, [8]. Jonathan Mills and his research group have been studying the antibiotic nature of natural product anaephene B. Anaephene A and B are alkyl phenolic natural products made by marine cyanobacteria (*Hormoscilla* sp., Oscillatoriales) with anaephene B containing a terminal alkyne functional group [9]. Mills’ group has synthesized analogues of anaephene B and found that some of them show antimicrobial activity when tested against methicillin-susceptible *Staphylococcus aureus* (MSSA) and methicillin-resistant *Staphylococcus aureus* (MRSA) [10,11]. In their study, they observed that certain structural changes, specifically the removal of the terminal alkyne in Anaephene B, the addition of an internal alkyne, the removal of the internal alkene, and the substitution of the phenol moiety with a 2-hydroxypyridine isostere enhanced drug performance [10,11]. Compounds **7** and **18** are most promising as they showed increased potency against MRSA and reduced hemolytic activity, respectively, compared to the natural product [10,11].

**Figure 1:**
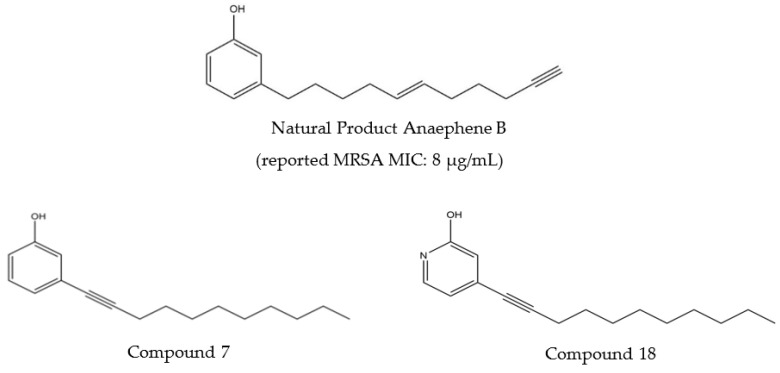
The three compounds tested in this study adapted from [10,11].

Subsequently, with the generous gift of the compounds by Jonathan Mills, we have now done experiments involving variations in test compound concentration so that potency against *Leishmania tarentolae* can be evaluated. The range of concentrations used for each compound was decided by referring to its minimum inhibitory concentration (MIC) against MRSA, as reported in Mills’ group’s previous studies involving the synthesis, characterization, and compound potency [10,11]. The MIC MRSA of each compound was used as a baseline, so the value is within or close to the range of concentrations used for each compound. All compounds tested with *Leishmania* were at concentrations between 0 and 12.2 µg/mL. Compounds in this study are numbered in accordance with Dr. Mills’ previous study [10,11]. The structures of natural product anaephene B and synthetic compounds **7** and **18** are shown in Figure 1.

## 2. Results and Discussion

### 2.1. Time of Addition Study and SAP Assay with Natural Product Anaephene B

**Figure 2:**
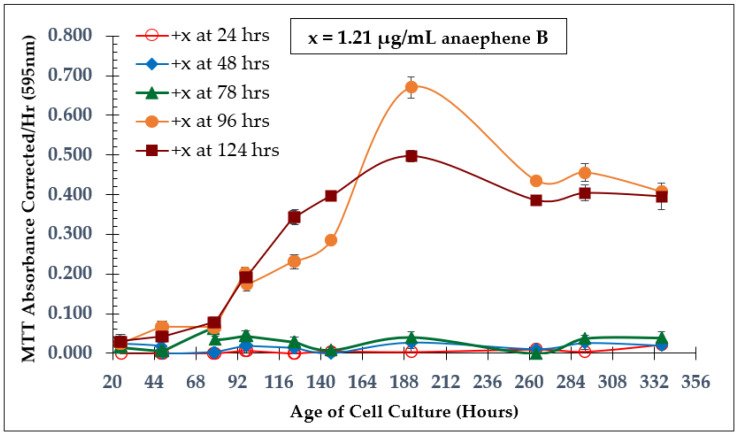
Time of Addition Study—Natural Product Anaephene B (1.21 µg/mL). Compound was added to each flask at different times to assess how time of addition affected the inhibition of *L. tarentolae*. Values are the mean and standard deviation for *n* = 4 replicates.

Incubation of *Leishmania tarentolae* with anaephene B was done to determine the approximate cell culture age at which test compound addition should occur for all dose-dependent experiments in this study. The same final concentration (1.21 µg/mL) of anaephene B was used for all flasks. The only difference between flasks is the cell culture age at which anaephene B was added. As shown in Figure 2, the time of addition affects the inhibition of *L. tarentolae,* specifically with regard to cell proliferation. From light microscopy, it was evident that cell abundance varied between the flasks in accordance with when compound addition occurred. Adding anaephene B at 24, 48, or 78 h resulted in complete inhibition of *L. tarentolae* with no recovery, demonstrating that early compound addition (preceding the log phase) is too detrimental for cell survival. Adding anaephene B at 96 or 124 h resulted in general growth curves with all phases identifiable (lag, log, stationary, and senescence). From this experiment, it was decided that compound addition should occur during the early-to-middle log phase of a cell culture’s growth curve for every dose-dependent experiment that was subsequently performed.

**Figure 3:**
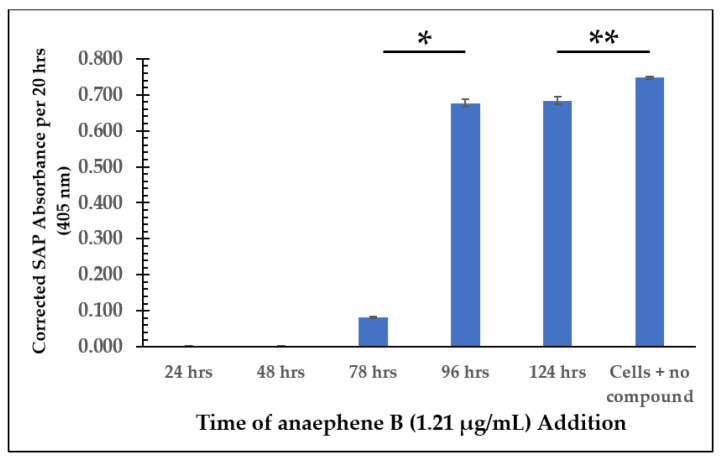
SAP Assay Following Time of Addition Study—Anaephene B Senescence Phase (Cell Culture Age: 528 h). Values are the mean and standard deviation for *n* = 3 replicates. * denotes *p* < 0.05 between addition at 78 h vs. 96 h. ** denotes *p* < 0.05 between addition at 124 h vs. no compound addition.

A secreted acid phosphatase (SAP) assay was performed using each flask from the time of addition study after all cell cultures reached the senescence phase. This was to ensure that the cells from each flask were able to secrete as many enzymes as possible into the culture medium throughout their lifetime. As shown in Figure 3, when anaephene B addition occurred at or before 48 h, there was little to no detectable SAP activity. In contrast, compound addition at or after 96 h resulted in much greater detectable SAP activity. This could mean that early compound addition (≤78 h) completely inhibits the ability of *L. tarentolae* to secrete acid phosphatases into their medium. Another explanation could be that early compound addition inhibits cell proliferation, which may result in there not being enough cells in the culture to secrete enough SAP for detectable activity. Thus, adding test compounds in the early-to-middle log phase rather than earlier (in the lag phase) is essential in allowing the cells to proliferate and secrete enough acid phosphatases to be detectable in the SAP assay.

### 2.2. Dose-Dependent Effect Studies and SAP Assays

**Figure 4:**
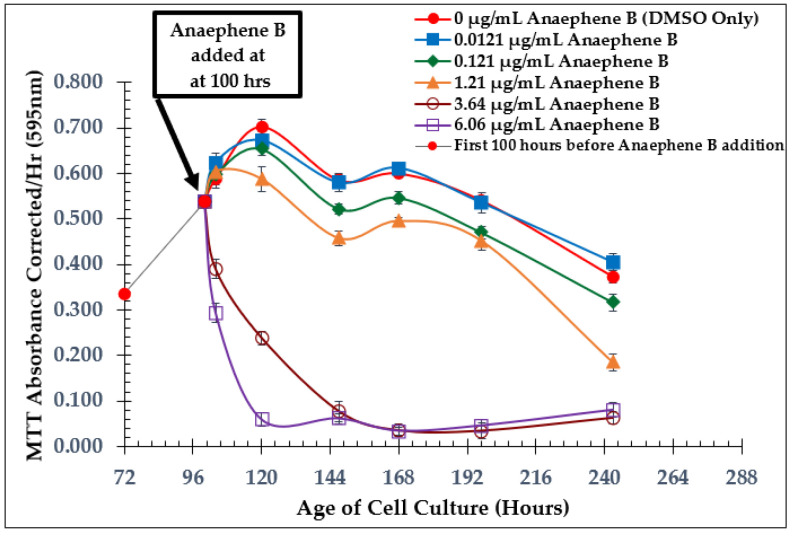
Dose-Dependent Effect of Natural Product Anaephene B on *L. tarentolae*. Additions at 100 h; Values are the mean and standard deviation for *n* = 4 replicates.

**Figure 5:**
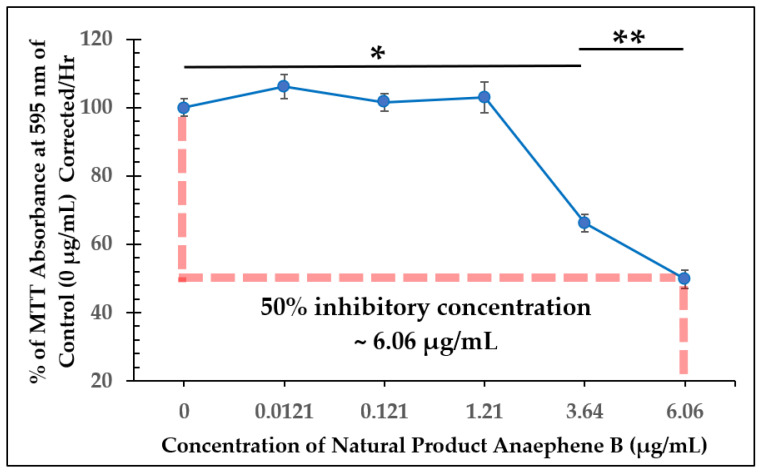
Dose-Dependent Effect of Anaephene B on *L. tarentolae* Immediately Following Addition (100 h). Values are the mean and standard deviation for *n* = 4 replicates. * denotes *p* < 0.05 between 0 µg/mL and 3.64 µg/mL of anaephene B. ** denotes *p* < 0.05 between 3.64 µg/mL and 6.06 µg/mL of anaephene B.

**Figure 6:**
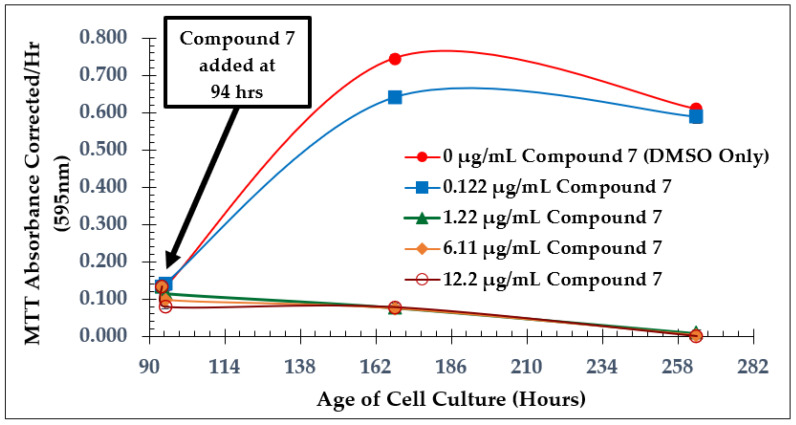
Dose-Dependent Effect of Compound **7** on *L. tarentolae*. Additions at 94 h; Values are the mean and standard deviation for *n* = 4 replicates; in some cases, the SD error bars are difficult to see since they are small relative to the circle or square size used.

**Figure 7:**
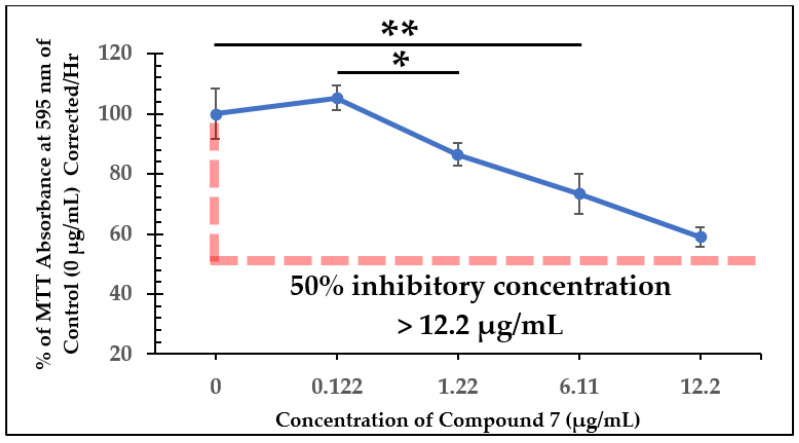
Dose-Dependent Effect of Compound **7** on *L. tarentolae* Immediately Following Addition (94 h). Values are the mean and standard deviation for *n* = 4 replicates. * denotes *p* < 0.05 between 0.122 µg/mL and 1.22 µg/mL of compound **7**. ** denotes *p* < 0.05 between 0 µg/mL and 6.11 µg/mL of compound **7**.

**Figure 8:**
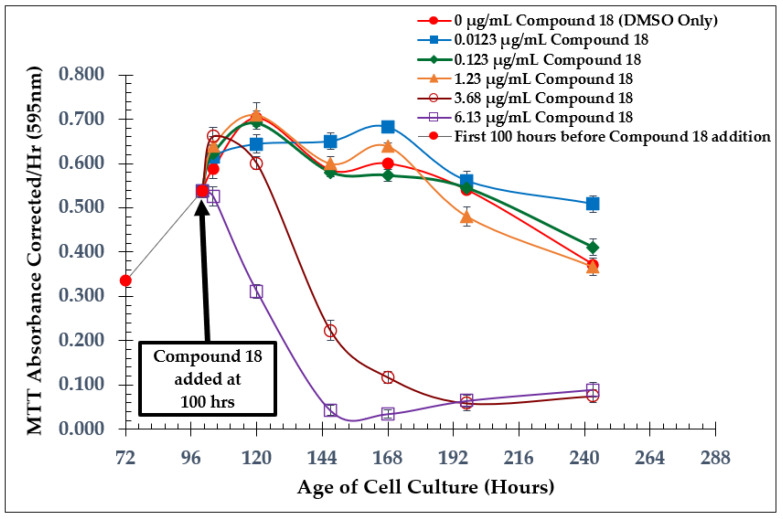
Dose-Dependent Effect of Compound **18** on *L. tarentolae*—Addition at 100 h; Values are the mean and standard deviation for *n* = 4 replicates.

**Figure 9:**
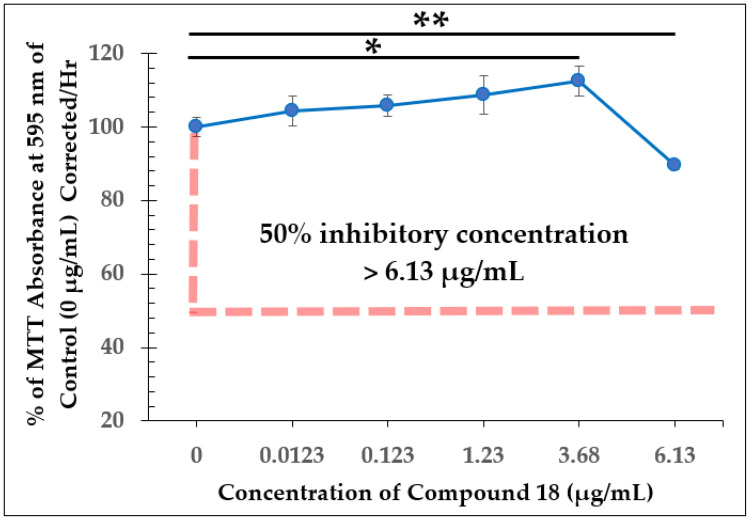
Dose-Dependent Effect of Compound **18** on *L. tarentolae* Immediately Following Addition (100 h). Values are the mean and standard deviation for *n* = 4 replicates. * denotes *p* < 0.05 between 0 µg/mL and 3.68 µg/mL of compound **18**. ** denotes *p* < 0.05 between 0 µg/mL and 6.13 µg/mL of compound **18**.

Growth curves from dose-dependent experiments for all three of the test compounds are shown in Figure 4, Figure 6, and Figure 8. *L. tarentolae* activity was monitored at various times following each compound addition. MIC values are reported in Table 1, and IC_50_ values were estimated from Figure 5, Figure 7, and Figure 9 and reported in Table 2. The MIC and IC_50_ values with the test compounds are very similar, indicating a narrow range between no disruption of viability and a substantial effect on viability.

Starting with the natural product (anaephene B), when ≥3.64 µg/mL of the compound was added to the cell culture at 100 h, *L. tarentolae* viability was substantially reduced, and the cells failed to recover afterward (Figure 4). Although the MIC using anaephene B against MRSA was found to be 8 µg/mL [10,11], the MIC value of anaephene B against *L. tarentolae* is at a lower concentration.

For compound **7**, the cells were completely inhibited with concentrations ≥ 1.22 µg/mL (Figure 6), yet 0.122 µg/mL of compound **7** did not show significant inhibition of the cells compared to the control group. In this experiment, it was observed that once a certain concentration threshold was reached, the cells were completely inhibited, showing no detectable viability. At the time of addition, however, no concentration used in the experiment had at least a 50% inhibitory effect on *L. tarentolae* (Figure 7). This suggests that the inhibitory effect of compound **7** may take longer to occur than that of the natural product. However, the MIC of this compound against MRSA was reported to be 2 µg/mL [10,11]. This indicates that the potency of the test compounds may change depending on the type of organism the compounds are incubated with.

For compound **18**, the structure is the same as compound **7** except for an alteration to the aromatic ring. A nitrogen atom replaces a methine group to make a pyridine, producing a heterocyclic compound with slightly different properties. Similar to the natural product, concentrations below 2 µg/mL did not cause a significant change in viability compared to control cells. However, when *L. tarentolae* cultures were treated with 3.68 µg/mL of compound **18**, a large decrease in reductase activity was observed, but this notably occurred after the cells seemed to have normal activity for the first 20 h after compound addition (Figure 8). Unlike how the natural product at 3.64 µg/mL completely inhibited *L. tarentolae* in just 47 h, compound **18** at 3.68 µg/mL caused gradual inhibition. As for the IC_50_ of compound **18**, none of the concentrations tested caused ≥ 50% inhibition (Figure 9), similar to what was found with compound **7**. With the MIC for MRSA of compound **18** at 8 µg/mL [10,11], it has become evident again that the IC_50_ of the test compounds against *L. tarentolae* may be unrelated to their MIC values against MRSA. However, the MIC for MRSA of each compound seems to be relatively similar to the concentration needed to cause gradual inhibition of *L. tarentolae*, as in the case of compound **18**.

As shown in Table 1, the MIC values for all three tested compounds are very similar, with compound **7** having a moderately lower value. The IC_50_ values in Table 2 indicate that the change in position of the alkyne group resulted in a modest loss of efficacy, while the replacement of a ring carbon with a nitrogen atom had limited effect relative to the parent compound.

**Figure 10:**
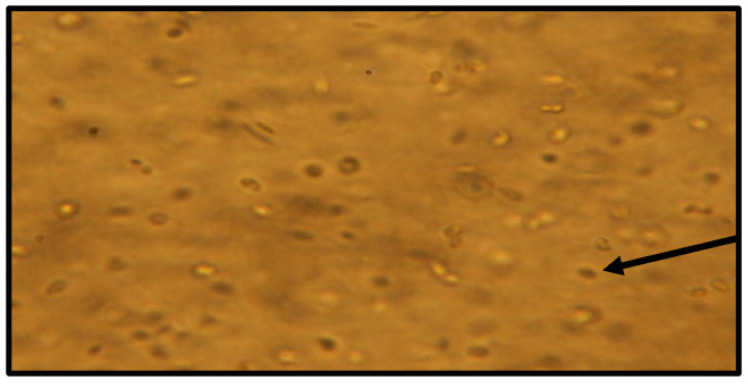
*L. tarentolae* treated with DMSO and no test compound (control cells) 143 h after treatment. 400 × magnification. Arrow indicates individual cells; we observed high cell abundance, elongated cells, flagella present, and normal motility.

**Figure 11:**
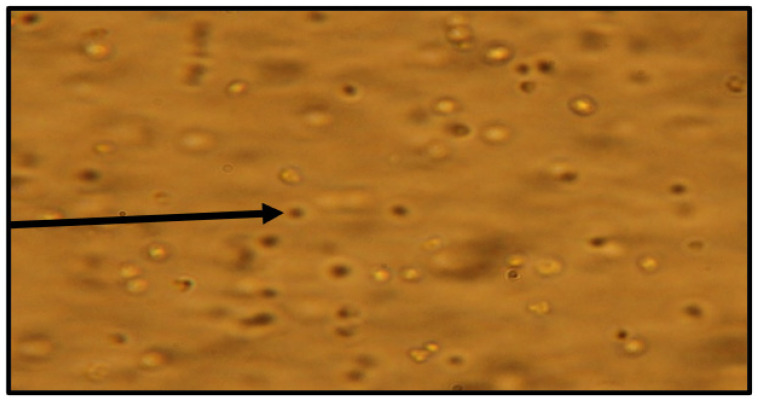
*L. tarentolae* treated with Compound **18** in DMSO (6.13 µg/mL) 143 h after treatment. 400× magnification. Arrow indicates individual cells; we observed low cell abundance, circular-shaped cells, flagella scarcity, and reduced motility.

When viewing cell cultures under light microscopy throughout the dose-dependent effect experiments (Figure 10 and Figure 11), high concentrations of the natural product and analogues caused fewer flagella to be seen projecting out from the cell bodies of *L. tarentolae* and less elongated (normal morphology) cells and more circular shaped cells. The motility of the cells in the presence of test compounds appeared substantially reduced. The visual changes in the cells seen in these microscopic images were similar for all compounds in this study. These findings suggest that anaephene B and its analogs are forcing *Leishmania* cells to revert to the amastigote form from the free-swimming promastigote form.

**Figure 12:**
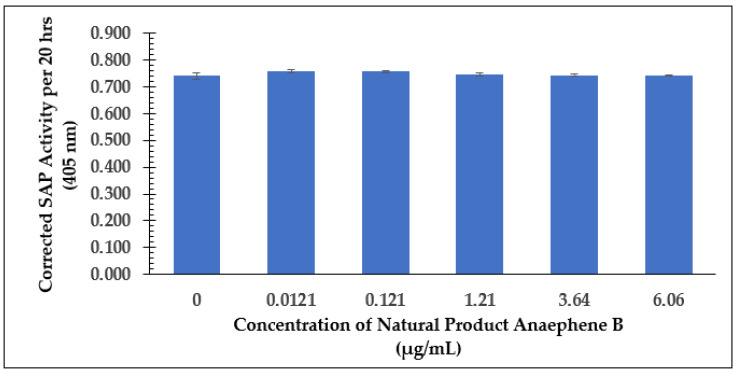
SAP Assay Following Dose-Dependent Study of Anaephene B—Senescence Phase (Cell Culture Age: 648 h). Values are the mean and standard deviation for *n* = 3 replicates.

**Figure 13:**
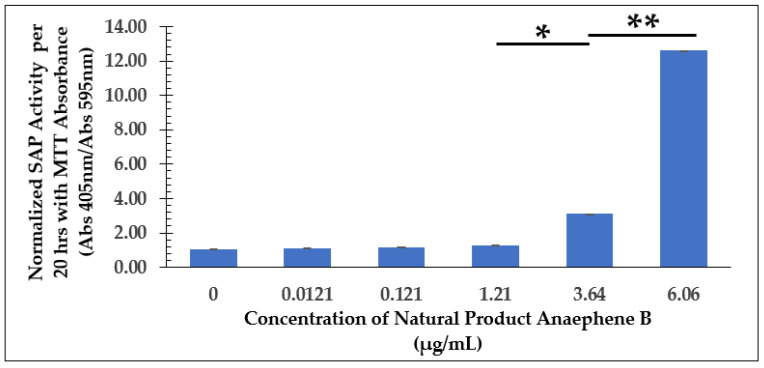
SAP Assay Following Dose-Dependent Study of Anaephene B—Senescence Phase (Cell Culture Age: 648 h) with SAP Enzyme Activity Normalized by MTT Cell Viability Data. Values are the mean and standard deviation for *n* = 3 replicates. * denotes *p* < 0.05 between 1.21 µg/mL and 3.64 µg/mL of anaephene B. ** denotes *p* < 0.05 between 3.64 µg/mL and 6.06 µg/mL of anaephene B.

For further examination, the SAP assay was performed following the dose-dependent experiments of the test compounds. As shown in Figure 12, the natural product did not result in a loss of secreted acid phosphatase activity as a function of the amount added, regardless of what concentration of the test compound was used. However, when the SAP enzyme activity was normalized by the cell viability data (from the same flasks), this ratio only changed at concentrations higher than 1.21 µg/mL, as shown in Figure 13.

## 3. Materials, Methods, and Procedures

### 3.1. Cell Cultures of L. tarentolae

In this current study, promastigotes of the parasitic reptile strain *Leishmania tarentolae* were cultured and used as a test model system [12]. *Leishmania tarentolae* (ATCC Strain 30143) axenic promastigote cells were cultured in brain heart infusion (BHI) medium with hemin and penicillin/streptomycin in the dark at room temperature (~26 °C) following the methods of Morgenthaler et al. [13]. Ten-milliliter stock cultures were grown in fifty-milliliter flasks and transferred every four days to new flasks containing fresh BHI medium to maintain stock cultures.

### 3.2. MTT Assay for Cell Viability

To assess the growth of the cells in varying test compound concentrations, cell viability tests were performed using the 3-[4,5-dimethylthiazol-2-yl]-2,5-diphenyltetrazolium bromide (MTT) assay following the procedure of Mossman [14]. Flatbottom 96-well plates were used for these assays, and the absorbance values were recorded at a wavelength of 595 nm using the Bio-Rad^®^ Microplate Reader Benchmark. Data were obtained from four replicates and reported as mean ± standard deviation.

### 3.3. Examining Dose-Dependent Effect of Test Compounds

To study the dose-dependent effect of test compounds on *L. tarentolae*, new ten-milliliter cell cultures were generated by using fresh BHI medium and adding a 2.0 mL aliquot from a stock cell culture into each new flask. A flask containing ten milliliters of only BHI medium (without *L. tarentolae*) was created for each experiment to serve as the blank. The new cell cultures were treated with specific concentrations of the test compounds in dimethyl sulfoxide (DMSO). The cell culture containing only DMSO (without any test compound) served as the experimental control. The final concentration of DMSO in each cell culture flask and the control flask was 1%. In this study, compound addition only occurred once for each culture so that the dose-dependent effect (short-term and long-term) could be evaluated.

The intensity of the inhibitory effect of a test compound on *L. tarentolae* may differ depending on when the single compound addition occurs during a culture growth curve. Therefore, to have consistency between experiments and be able to effectively cross-analyze results, a preliminary experiment was performed to help guide the decision of what approximate cell culture age all compound additions in this study should occur. Since anaephene B is the parent compound of the synthetic analogs being studied, it was the only compound used for this preliminary experiment. Results obtained using anaephene B were used to determine the age of cell culture; every other test compound addition should occur. Only one concentration of anaephene B (1.21 µg/mL)., specifically one that is below its MIC, was used for this time of addition study.

Cell viability data were obtained for all flasks using the MTT assay [14] by taking samples from each flask daily. On the day of compound addition, the MTT assay assessed culture flask cell viability both before and after addition so that the immediate dose-dependent effect of each compound could be evaluated. Cell viability data from the day of addition were used to estimate the 50% inhibitory concentrations (IC_50_) of each compound. The long-term dose-dependent effect of each test compound was examined by generating growth curves using the cell viability data obtained from each day. For each compound, growth curves of experimental flasks (treated cells) were analyzed for any significant changes from the growth curve of the control flask (untreated cells). Additionally, light microscopy was used throughout this study to observe any changes in the shape, motility, and clumping of the cells with and without treatment of the test compounds.

### 3.4. Examining SAP Activity from L. tarentolae

*L. tarentolae* are known to secrete enzymes into their medium in culture [15]. One class of these enzymes, the secreted acid phosphatases (SAP), is reported to assist the parasitic cells in infecting host macrophages [16,17]. If a test compound can inhibit SAP activity from *L. tarentolae*, then the compound may help limit the infection by these parasitic cells, therefore making it a possible component of a new therapy for leishmaniasis. For this study, it was relevant to compare the SAP activity of flasks that contain different concentrations of test compounds. Therefore, enzyme assays were performed (using the method of Mendez et al., [18] using samples from the same flasks that were used for the dose-dependent effect experiments.

To prepare the assay, samples of *L. tarentolae* and BHI only (control) were collected in two-milliliter microcentrifuge tubes from the experimental flasks of interest. The samples were then centrifuged (10,000× *g*, 5 min) to separate the cells from the medium. The supernatant was collected from these samples and placed in new microcentrifuge tubes to make uniform pools. The supernatant from each flask sample was then distributed equally into three microcentrifuge tubes for *n* = 3 replicates (450 µL each). SAP activity was evaluated using para-nitrophenyl phosphate (*p*NPP) as the substrate in 0.5 M sodium acetate buffer at pH 4.5. Into each replicate tube containing 450 µL of supernatant, 450 µL of sodium acetate buffer was added. A separate tube was also created to serve as the blank for the assay. The blank consisted of 900 µL of sodium acetate buffer instead of 450 µL of buffer and 450 µL supernatant/BHI. Once all sample tubes were ready, the enzyme reaction was started by adding 100 µL of substrate (5 mg *p*NPP/1 mL 0.5 M sodium acetate buffer pH 4.5: 1.75 mM *p*NPP final concentration) into each sample tube. The tubes were then stored in the dark for an incubation time of 20 h. After this time had passed, the assay was stopped by adding 100 µL of 10 M NaOH into each sample tube. The samples were then measured individually in a spectrophotometer at a wavelength of 405 nm. The absorbance values were recorded and then graphed to determine whether a test compound has a concentration-dependent effect on SAP activity. A higher absorbance value in one sample compared to another sample indicates more relative SAP activity in the first sample.

The SAP enzyme assay was performed following the time of addition study for the natural product anaephene B as well as after the dose-dependent effect experiments of those test compounds that caused slow or gradual inhibition of *L. tarentolae* instead of complete inhibition at certain concentrations. For comparison purposes, the SAP assay was also performed following a dose-dependent experiment of the natural product, even though complete inhibition is observed, as discussed in Section 3.2. SAP activity was quantified and compared between samples using the ratio of the absorbance value obtained from the SAP assay to the absorbance value obtained from the MTT assay that was performed during the stationary phase of the cell culture growth curve.

### 3.5. Data Collection and Analysis

Absorbance values of cell flasks obtained with the MTT assay were corrected by subtracting off the absorbance value of the blank (flask with only BHI medium and 1% DMSO). These new values were then adjusted to reflect one hour of incubation time. Values are reported and graphed as mean ± standard deviation for *n* = 4 replicates in the MTT assays, and *n* = 3 replicates in the SAP enzyme assays. Values between different groups of data were evaluated for statistical significance using one-way ANOVA, where a *p* < 0.05 is considered sufficient evidence for a significant difference.

## 4. Conclusions

This work has now expanded the list of natural products that can negatively affect *Leishmania* viability in culture. Our results indicate that the anaephene B natural product evaluated in this study, along with two synthetic analogs, are effective at levels similar to that of meglumine antimoniate and amphotericin B, two drugs currently used in the therapy of *Leishmania* infections [12]. It is of interest that anaephene effects on viability were observed shortly after addition at concentrations above the IC_50_ value. All three test compounds were equally or more effective at inhibition of *Leishmania tarentolae* viability than against MRSA (as reported as 2–8 µg/mL by Kukla et al., [10,11]. The test compounds had very limited effect on detectable secreted acid phosphatase activity (SAP); thus, this natural product may not change the potential infectivity of parasites that are not inhibited by this class of potential drugs. Clearly, the mechanism of action of the anaephene class of compounds needs to be determined.

## Figures and Tables

**Table 1:** Minimum Inhibitory Concentrations (MIC) Observed for Anaephene B and Analogs Against *L. tarentolae*.

Test Compound	Minimum Inhibitory Concentration (MIC)
Natural Product (Anaephene B)	~3.64 µg/mL
Compound 7	~1.22 µg/mL
Compound 18	~3.68 µg/mL

**Table 2:** IC_50_ Values for Anaephene B and Analogs Against *L. tarentolae*.

Test Compound	50% Inhibitory Concentration (IC_50_)
Natural Product (Anaephene B)	~6.06 µg/mL
Compound 7	>12.2 µg/mL
Compound 18	>6.13 µg/mL

## Data Availability

Data are all provided in the manuscript.
